# Altitudinal variation of dragon fruit metabolite profiles as revealed by UPLC-MS/MS-based widely targeted metabolomics analysis

**DOI:** 10.1186/s12870-024-05011-w

**Published:** 2024-04-29

**Authors:** Zhibing Zhao, Lang Wang, Jiajia Chen, Ni Zhang, Wei Zhou, Yuehua Song

**Affiliations:** 1 School of Karst Science, Guizhou Normal University/ State Engineering Technology Institute for Karst Desertification Control, Guiyang, 550001 China; 2 College of Food Science and Engineering, Guiyang University, Guiyang, 550003 China; 3 Guizhou Institute of Soil and Fertilizer, Guiyang, 540086 China

**Keywords:** Pitaya (dragon fruit), Production area, Altitude, Fruit quality, Metabolic profiling, LC-MS/MS

## Abstract

**Background:**

Geographical factors affect the nutritional, therapeutic and commercial values of fruits. Dragon fruit (*Hylocereus spp*) is a popular fruit in Asia and a potential functional food with diverse pharmacological attributes. Although it is produced in various localities, the information related to the altitudinal variation of dragon fruit nutrients and active compounds is scarce. Hence, this study aimed to investigate the variations in metabolite profiles of *H. polyrhizus* (variety Jindu1) fruit pulps from three different altitudes of China, including Wangmo (WM, 650 m), Luodian (LD, 420 m), and Zhenning (ZN, 356 m). Jindu1 is the main cultivated pitaya variety in Guizhou province, China.

**Results:**

The LC-MS (liquid chromatography-mass spectroscopy)-based widely targeted metabolic profiling identified 645 metabolites, of which flavonoids (22.64%), lipids (13.80%), phenolic acids (12.40%), amino acids and derivatives (10.39%), alkaloids (8.84%), and organic acids (8.37%) were dominant. Multivariate analyses unveiled that the metabolite profiles of the fruit differed regarding the altitude. Fruits from WM (highest altitude) were prime in quality, with higher levels of flavonoids, alkaloids, nucleotides and derivatives, amino acids and derivatives, and vitamins. Fruits from LD and ZN had the highest relative content of phenolic acids and terpenoids, respectively. We identified 69 significantly differentially accumulated metabolites across the pulps of the fruits from the three locations. KEGG analysis revealed that flavone and flavonol biosynthesis and isoflavonoid biosynthesis were the most differentially regulated. It was noteworthy that most active flavonoid compounds exhibited an increasing accumulation pattern along with the increase in altitude. Vitexin and isovitexin were the major differentially accumulated flavonoids. Furthermore, we identified two potential metabolic biomarkers (vitexin and kaempferol 3-O-[2-O-β-D-galactose-6-O-a-L-rhamnose]-β-D-glucoside) to discriminate between dragon fruits from different geographical origins.

**Conclusion:**

Our findings provide insights into metabolic changes in dragon fruits grown at different altitudes. Furthermore, they show that growing pitaya at high altitudes can produce fruit with higher levels of bioactive compounds, particularly flavonoids.

**Supplementary Information:**

The online version contains supplementary material available at 10.1186/s12870-024-05011-w.

## Background

Foods, including fruits, vegetables, dairy products, etc., are vital for human health, as they are involved in the management, treatment and prevention of diseases [1, 2]. Among these, fruits are important sources of numerous essential nutraceuticals [3]. The daily intake of fruits is recommended for a well-balanced, healthy diet enriched with bioactive metabolites for the prevention of several chronic diseases [3, 4]. An adequate daily intake of vegetables and fruits substitutes high-energy-dense foods from the body and promotes the ingestion of dietary fiber and healthy nutrients [5, 6]. The recommended daily consumption of fresh fruits and vegetables by the WHO (World Health Organization) is at least 400 g [7]. Therefore, understanding fruit plants’ biology and factors underlying variation in fruits’ quality traits is of great interest. Studies have shown that environmental factors, such as altitude, light, humidity, temperature, atmospheric gases, and rootstocks, affect the normal growth, development and reproduction of fruit plants, leading to significant changes in the physical and biochemical characteristics of fruits [4, 8, 9]. For instance, the influence of different growing conditions on apple, strawberry, watermelon, peach, blackcurrant, goji berry and wolfberry quality traits has been studied and proven [8, 10–15].

Amongst the environmental factors, altitude is a major determinant of fruit quality, as it affects the temperature, solar radiation (UV light and light intensity), and humidity, leading to significant physiological and metabolic changes in plants [16–21]. Studies in strawberry, peach, and apple have shown that high altitude affects fruit color traits, stimulates the accumulation of antioxidation compounds, increases the acidity of fruits, induces variation in carotenoid profiles, impairs vitamin C synthesis, and alters protein metabolism [20–23]. For instance, an increase in altitude was associated with enhanced biosynthesis and storage of phenolic compounds, including quercetin-3-O-rhamnoside, cyanidin-3-O-galactoside, chlorogenic acid, and quercetin-3-O-rutinoside in apple [21]. Hence, evaluating the physicochemical profiles of fruits from different altitudes will provide valuable resources for the environmental-based production of fruits for specific industries and purposes.

Dragon fruit (pitaya or pitahaya) is an economically important tropical fruit of the *Cactaceae* family [24]. Three categories of dragon fruits, including red pulp with pink peel (*Hylocereus polyrhizus*), white pulp with pink peel (*H. undatus*), and white pulp with yellow skin (*H. megulanthus*) are found based on the peel and pulp colors [25]. Its higher adaptability, tolerance to various abiotic stresses, commercial interest and health-promoting attributes are some of the characteristics that have led to its cultivation all over the world [24, 26, 27]. Dragon fruits are rich in phytochemicals, such as betacyanin, lycopene, vitamins (vitamin C mainly), dietary fiber, flavonoids, amino acids, phenolic acids, sugars and organic acids [25, 28–31]. Pharmacological investigations have shown that dragon fruit extracts have many therapeutic abilities, including anti-microbial, antioxidant, anti-cancer, anti-diabetic, anti-plasmodial, hypolipidemic, anti-inflammatory, chemopreventive, neuroprotective and anti-ulcer [25, 28, 32–40]. Betacyanin and peel powder from dragon fruit are used as natural colorants in food products or to improve food quality [28, 41, 42]. Variation in the content of nutraceuticals in dragon fruits is primarily governed by genotype, environmental conditions, and their interactions [37, 43, 44]. However, few studies have focused on the variation in quality characteristics of dragon fruits from different geographical origins [37, 43].

Pitaya is the fifth most popular tropical fruit in Asia [24]. The major pitaya producing areas in China are in the Southern, principally in Guangdong, Guizhou, and Guangxi provinces. Dragon fruit has been grown in Guizhou since about 2000. Its typical karst landforms, humid tropical climate, annual precipitation (1100–1335 mm) and annual average temperature (14–16 °C) are the most suitable for growing dragon fruit [24, 45]. The planted area and annual output of pitaya in Guizhou are about 6,670 hectares (110,388 acres) and 50,000 tones, respectively. Three counties, namely Wangmo, Luodian and Zhenning are the top planting locations in Guizhou province (Figure S1). The total planted area and production in the three counties by 2019 were 670 hectares (11,089 acres) and 7,000 tones, respectively. The three counties are primarily different in altitudes, offering the opportunity to investigate the altitudinal variation of dragon fruit phytochemical composition and quality traits. Understanding the impacts of altitude on dragon fruit quality values will contribute to meeting specific consumer demands and promoting the fruit industry.

In the last decade, metabolomics has emerged as the most powerful and efficient tool to chemically characterize (qualitatively and quantitatively) the metabolome underpinning the diversity in plant phenotypes and to evaluate metabolic changes due to environmental factors [46, 47]. It has been applied to investigate differences in the metabolite profiles of *Lycium barbarum* (Chinese wolfberry) fruits from three different locations in China [14]. Therefore, in this study, we investigated changes in the metabolite profiles of Jindu1 fruits (the major pitaya cultivar) from the three top growing areas of Guizhou provinces through widely targeted metabolomics analysis. We uncovered key DAMs (differentially accumulated metabolites) and differently regulated pathways between the three locations. Notably, we identified the bioactive compounds whose accumulation was influenced by growing conditions, mainly the altitude. Our findings provide fundamental resources for the environmental-based production of dragon fruits of desired quality.

## Results

### Metabolite profiles of dragon fruits from different altitudes

**Fig. 1:**
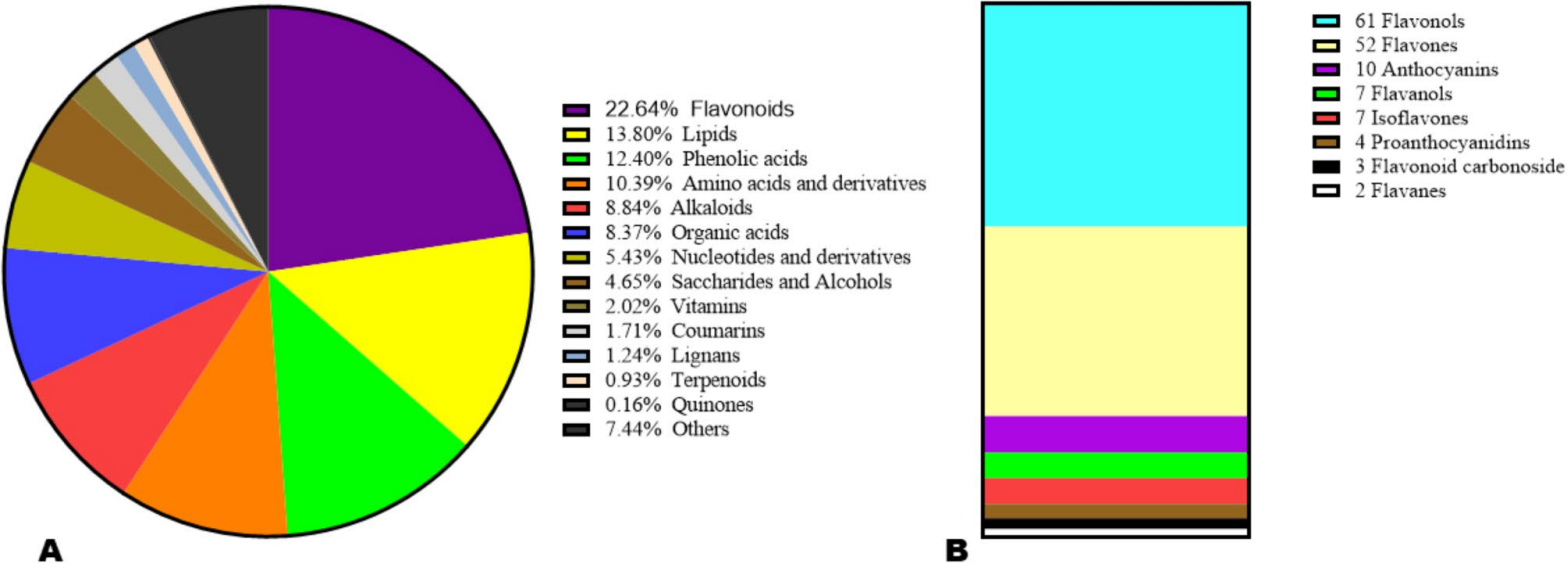
Classification of the identified metabolites. (**A**) Classification of the 645 identified metabolites. (**B**) Specific classification of flavonoid groups

**Fig. 2:**
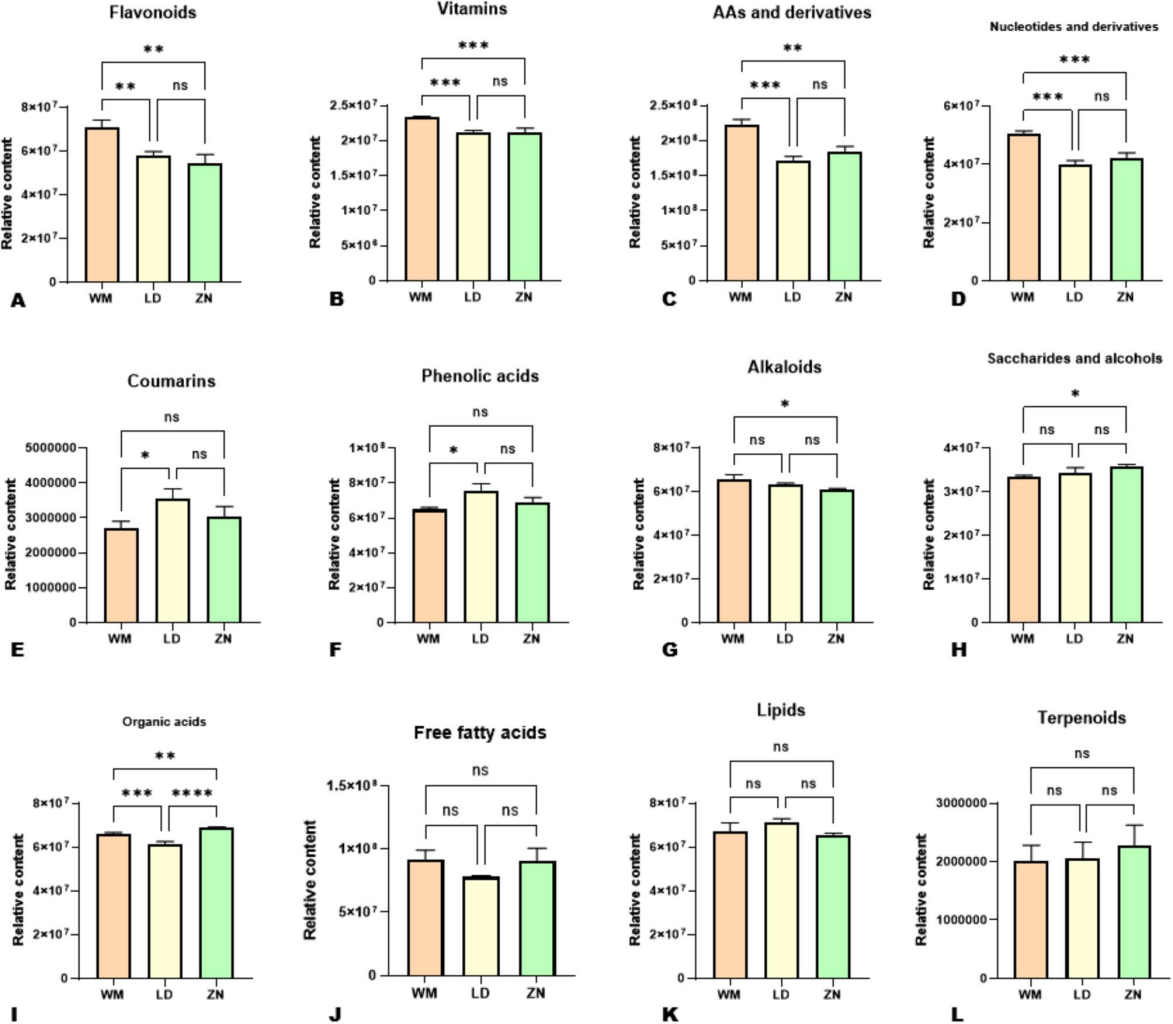
(**A**)-(**L**)Variation in the relative content of the major classes of metabolites in the fruits from the three different altitudes. The specific metabolite class is indicated at the top of each graph. ns, no significant difference. *, **, ***, and **** indicate significant differences at *P* ˂ 0.05, 0.01, 0.001, and 0.0001, respectively. WM, Wangmo County; LD, Luodian County; ZN, Zhenning County

To provide deep insights into the impacts of different altitudes on dragon fruit quality variation, we carried out the metabolic profiling analysis of fruits from the three locations. The UPLC-ESI-QqQLIT-MS/MS-based widely targeted metabolomics analysis approach was achieved on fruits from WM (650 m), LD (420 m), and ZN (356 m) (Figure S1) with three replications. All fruit extracts were analyzed at both the negative and positive ESI (electrospray ionization) modes. The higher correlations obtained between quality control (QC) samples confirmed the repeatability of the experiment (Figure S2). In total, 645 metabolites were detected and chemically characterized (TableS1). The classification of the metabolites revealed that flavonoids (22.64%), lipids (13.80%), phenolic acids (12.40%), amino acids and derivatives (10.39%), alkaloids (8.84%), and organic acids (8.37%) were the predominant metabolic compounds in the pulp of dragon fruit (Fig. 1A). The flavonoids were dominated by flavonols (41.78%) and flavones (35.61%) (Fig. 1B). The sum of all intensities of metabolites belonging to the same class showed that fruits from WM had significantly higher relative content of flavonoids, vitamins, amino acids and derivatives, and nucleotides and derivatives (Fig. 2A-D). Compared with fruits from WM, fruits from LD exhibited significantly higher relative content of coumarins and phenolic acids (Fig. 2E, F). The alkaloid content of fruits from WM was significantly higher than in fruit from ZN (Fig. 2G). In contrast, fruit from ZN had significantly higher relative content of saccharides and alcohols than fruits from WM (Fig. 2H). Fruit from ZN exhibited the highest relative content of organic acids, followed by fruits from WM (Fig. 2I). There were no significant differences in the relative contents of free fatty acids, lipids, and terpenoids in the fruits from the three locations (Fig. 2J-L).

### Altitudinal distribution and variation of dragon fruit metabolite profiles

**Fig. 3:**
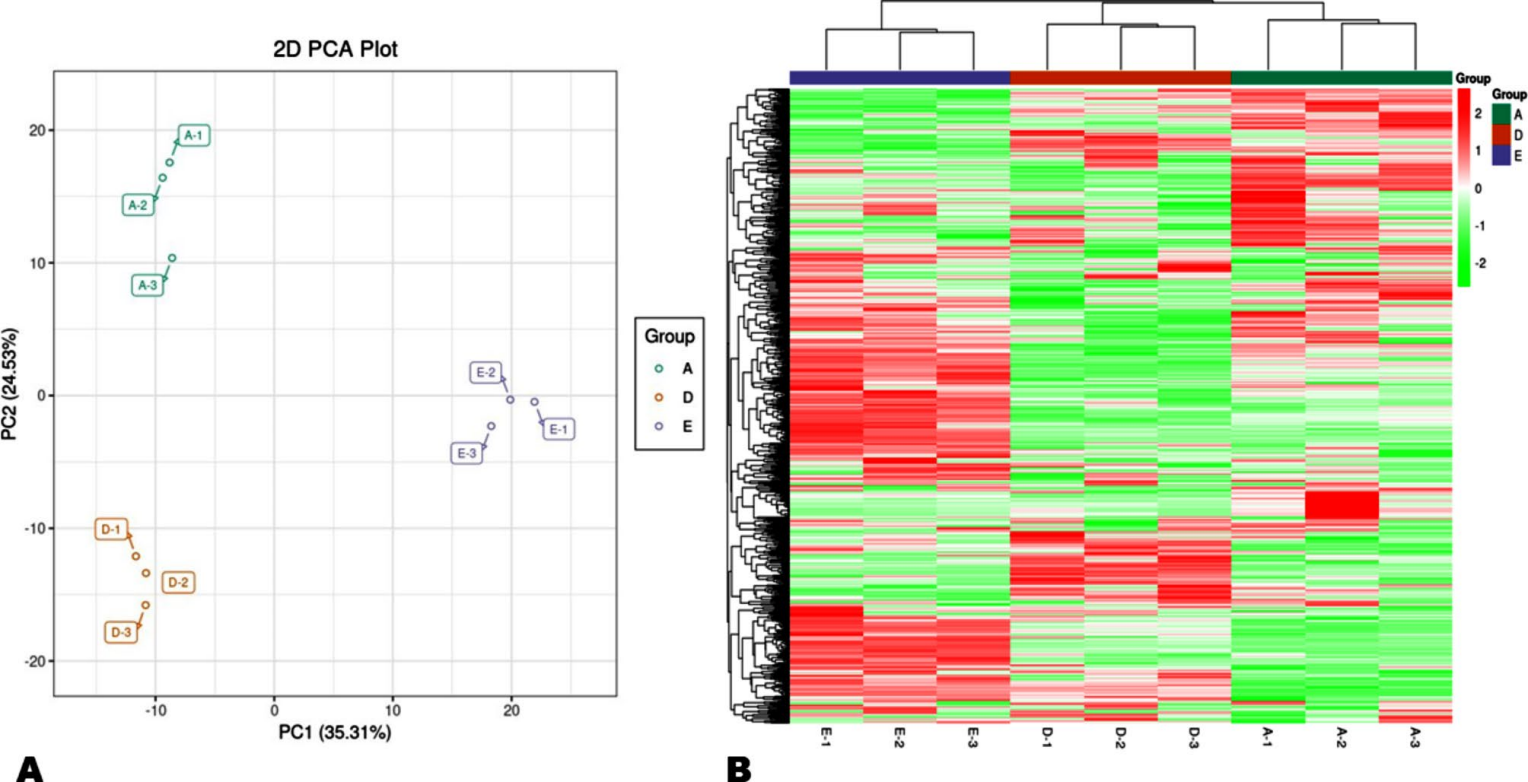
Overview of metabolite profiles of dragon fruits from the three different altitudes. (**A**) Principal component analysis (PCA). (**B**) Heat map visualization. Each sample is represented by one column, and each metabolite is visualized in one row. Red and green indicate relatively high and low metabolite abundance, respectively. **A**, **D**, and **E** indicate ZN (Zhenning County), LD (Luodian County), and WM (Wangmo County), respectively. 1, 2, and 3 refer to replications

**Fig. 4:**
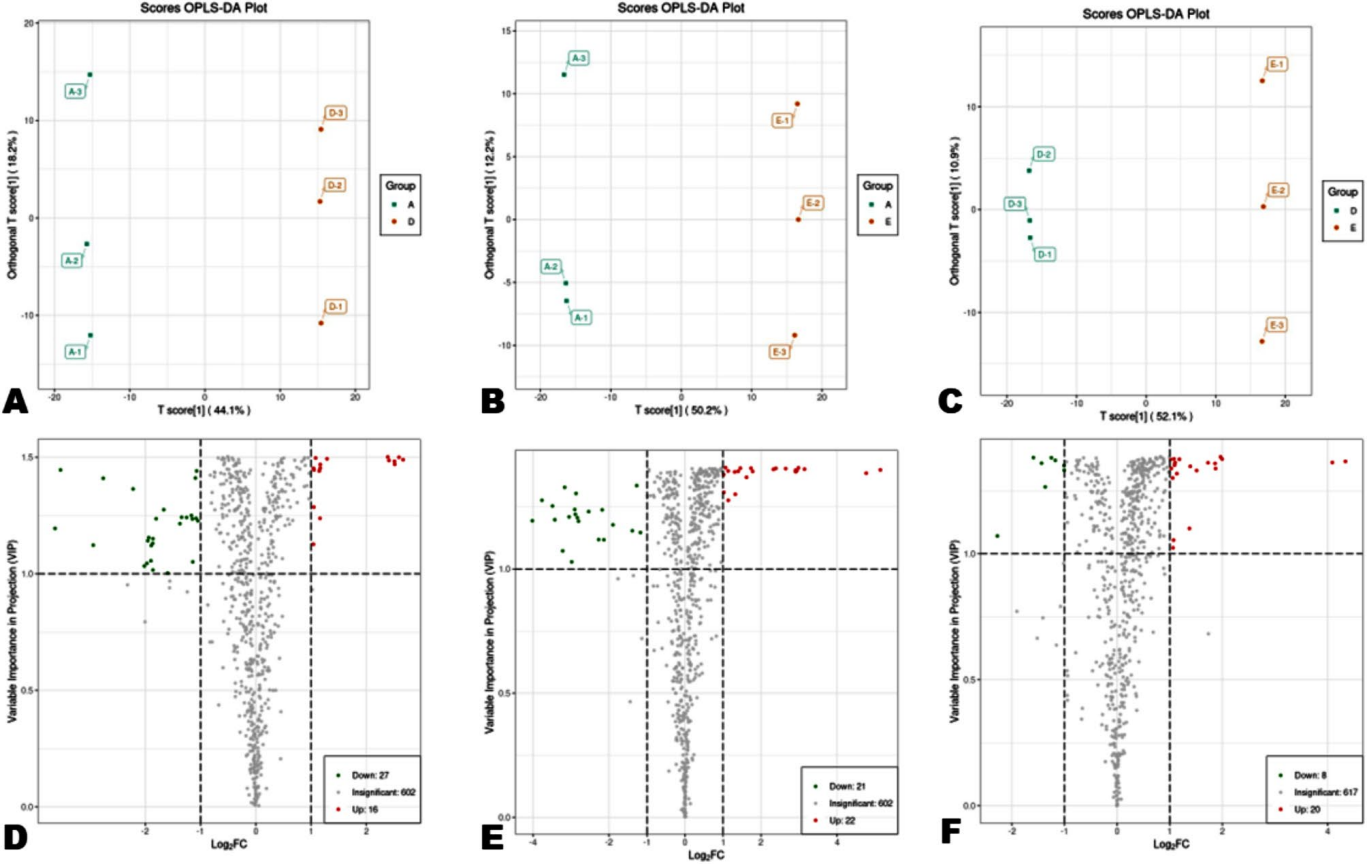
Pairwise comparison and differentially accumulated metabolites (DAMs). (**A**)-(**C**) OPLS-DA score plots of pairwise comparisons between ZN_vs_LD, ZN_vs_WM, and LD_vs_WM, respectively. (**D**)-(**F**) Volcano plots of DAMs in pairwise comparisons between ZN_vs_LD, ZN_vs_WM, and LD_vs_WM, respectively. WM, Wangmo County; LD, Luodian County; ZN, Zhenning County

We conducted PCA (principal component analysis) and HCA (hierarchical clustering analysis) analyses to examine the degree of metabolite variation in dragon fruits from different altitudes (Fig. 3). The PCA showed that the metabolite profiles of WM, LD, and ZN fruit samples were different and could be discriminated by PC1 (35.31%) and PC2 (24.53%) (Fig. 3A). Supportively, the HCA showed that a number of metabolites exhibited differential accumulation patterns in the pulp of the fruits from the three locations (Fig. 3B). Many metabolites exhibited higher accumulation patterns in fruits from WM compared with fruit from the two other locations. The results from correlation analysis were also supportive of the observed trends of metabolites’ variation in the fruits from different altitudes (Figure S2). Further, we performed OPLS-DA (orthogonal partial least squares-discriminant analysis) to verify the altitudinal variation of metabolites in dragon fruits. Supportively, the OPLS-DA indicated strong goodness of fit and high predictability when comparing the groups against each other (Figures S3)A-C). As shown in the score plots of OPLS-DA, the metabolite profile of fruits from ZN showed 44.1% and 50.2% variance when compared to fruits from LD and WM, respectively (Fig. 4A, B). Meanwhile, fruits from LD and WM showed 52.1% of variation (Fig. 4C).

### Differentially accumulated metabolites (DAMs) across the dragon fruits from different altitudes and KEGG analysis

**Fig. 5:**
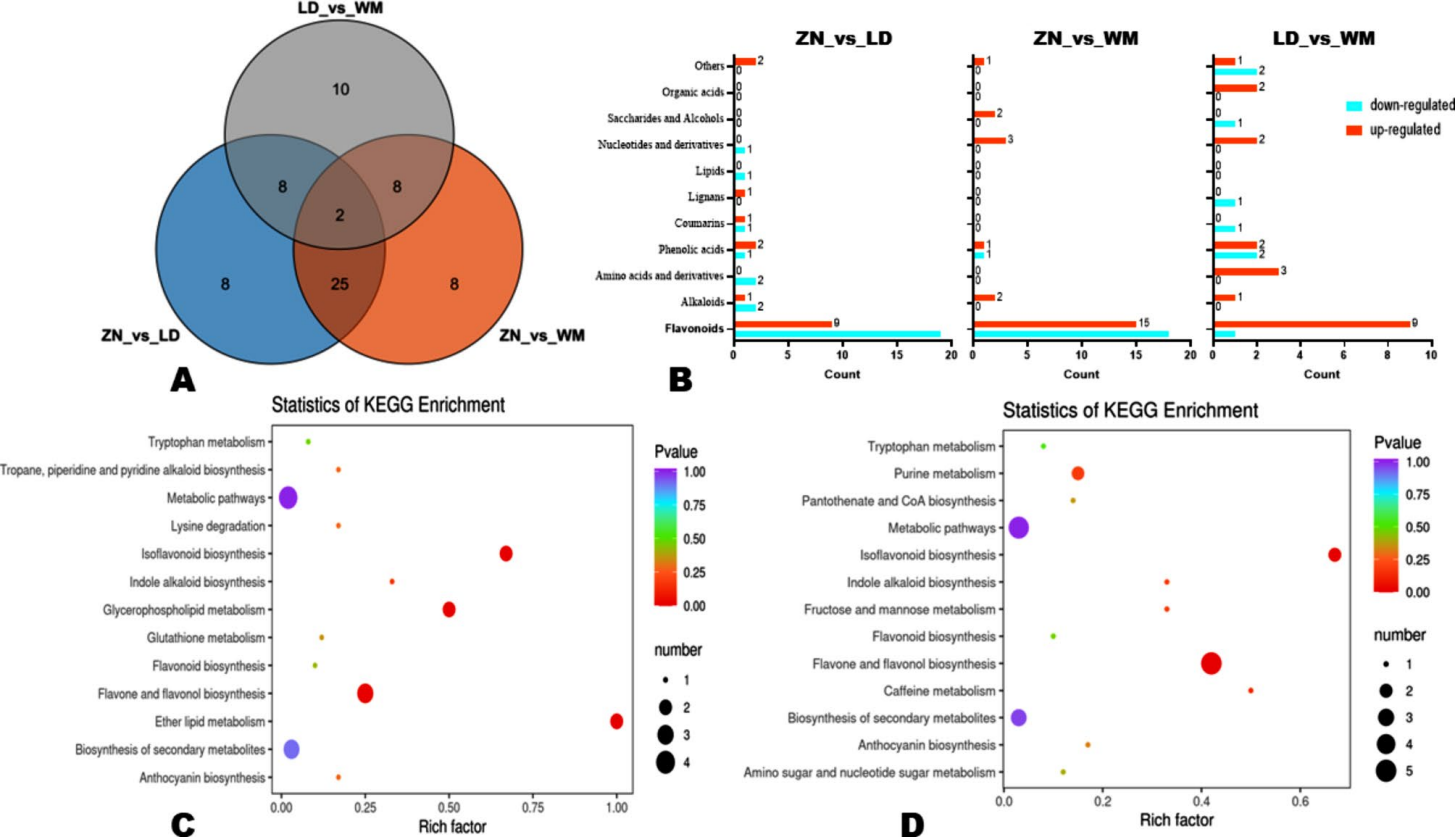
Overlapped DAMs and KEGG analysis. (**A**) Venn diagram showing the number of overlapped DAMs. (**B**) Classification of DAMs in each pairwise comparison. (**C**) and (**D**) KEGG annotations and enrichment results of the DAMs between ZN_vs_LD and ZN_vs_WM, respectively. WM, Wangmo County; LD, Luodian Country; ZN, Zhenning Country

To unravel the real impact of altitude on metabolites’ accumulation in dragon fruit, we carried out DAMs analysis. By applying the thresholds of fold-change (FC ≥ 1), VIP ≥ 1, and *p*-value < 0.05, we detected 43, 43, and 28 significant DAMs in pairwise comparison between ZN_vs_LD, ZN_vs_WM, and LD_vs_WM, respectively (Fig. 4D-F). Key DAMs could serve as discriminatory metabolic biomarkers. Through the construction of a Venn diagram among the DAMs, we identified two key DAMs, including Apigenin-8-C-Glucoside (Vitexin, a flavonoid carbonoside) and Kaempferol 3-O-[2-O-*β*-D-galactose-6-O-*a*-L-rhamnose]-*β*-D-glucoside (flavonol) (Fig. 5A). The classification of DAMs showed that flavonoids were the main metabolite class affected by the differences in altitudes (Fig. 5B).

To unveil the main metabolic pathways significantly affected by the growing conditions, we performed KEGG annotation and enrichment analysis of the DAMs. Supportively to the DAMs classification, the results indicated that flavone and flavonol biosynthesis and isoflavonoid biosynthesis were the most enriched pathways (Fig. 5C, D, and Figure S4). Glycerophospholipid metabolism and ether lipid metabolism were also significantly enriched between ZN and LD (Figure 5C).

### Variation of active flavonoid compounds

**Fig. 6:**
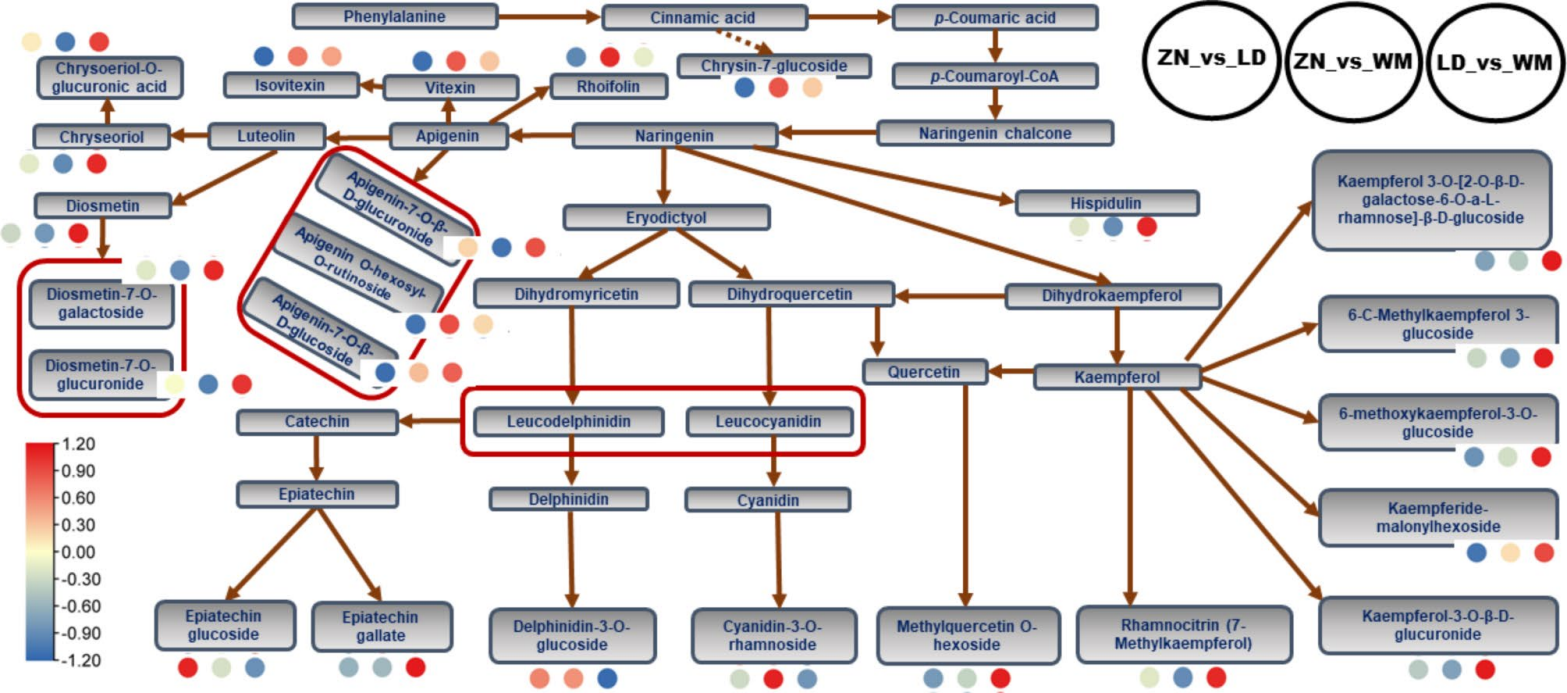
A diagram of the flavonoid biosynthesis pathway with highlights on major differentially accumulated bioactive flavonoid compounds. The scaled Log2 fold change of each metabolite is plotted in a blue-yellow-red color scale. The small red circles indicate the highest accumulation. WM, Wangmo County; LD, Luodian County; ZN, Zhenning County

To examine the variation characteristics of DAMs in fruits from WM, LD, and ZN, we first carried out K-means analysis of DAMs. The results showed that the DAMs were grouped into six sub-classes based on their relative contents (Figure S5 and Table S2). DAMs in sub-class 5 (13 metabolites) and sub-class 6 (19 metabolites), mostly flavonoids, exhibited the highest relative content in fruits from WM (Figure S5 and Table S2). Only eight metabolites in sub-class 2 exhibited the highest relative content in fruits from LD (Figure S5 and Table S2). As both the classification, KEGG and K-means analyses revealed flavonoids as the most affected by changes in altitude, we constructed a diagram of the flavonoid pathway and a heatmap based on the fold changes to favorize future exploration and identification of differentially expressed genes (Figure 6 and S6). Many active flavonoid compounds, including vitexin, isovitexin, diosmetin, hispidulin, rhoifolin, apigenin glucosides, diosmetin glucosides, epicatechin gallate, ferrerol-7-*O*-glucoside, kaempferol glucosides, pratensein-7-*O*-glucopyraniside, baicalin, etc., were highly accumulated in fruits from WM compared to those from LD and ZN (Figure 6 andS6). Genkwanin, oroxylin, acacetin, wogonin and biochanin A were highly induced in fruits from WM, followed by LD and ZN (Figure S6). Epicatechin glucoside had the highest relative content in fruits from LD, followed by WM (Figure 6 and S6).

### Variation characteristics of other DAMs

**Fig. 7:**
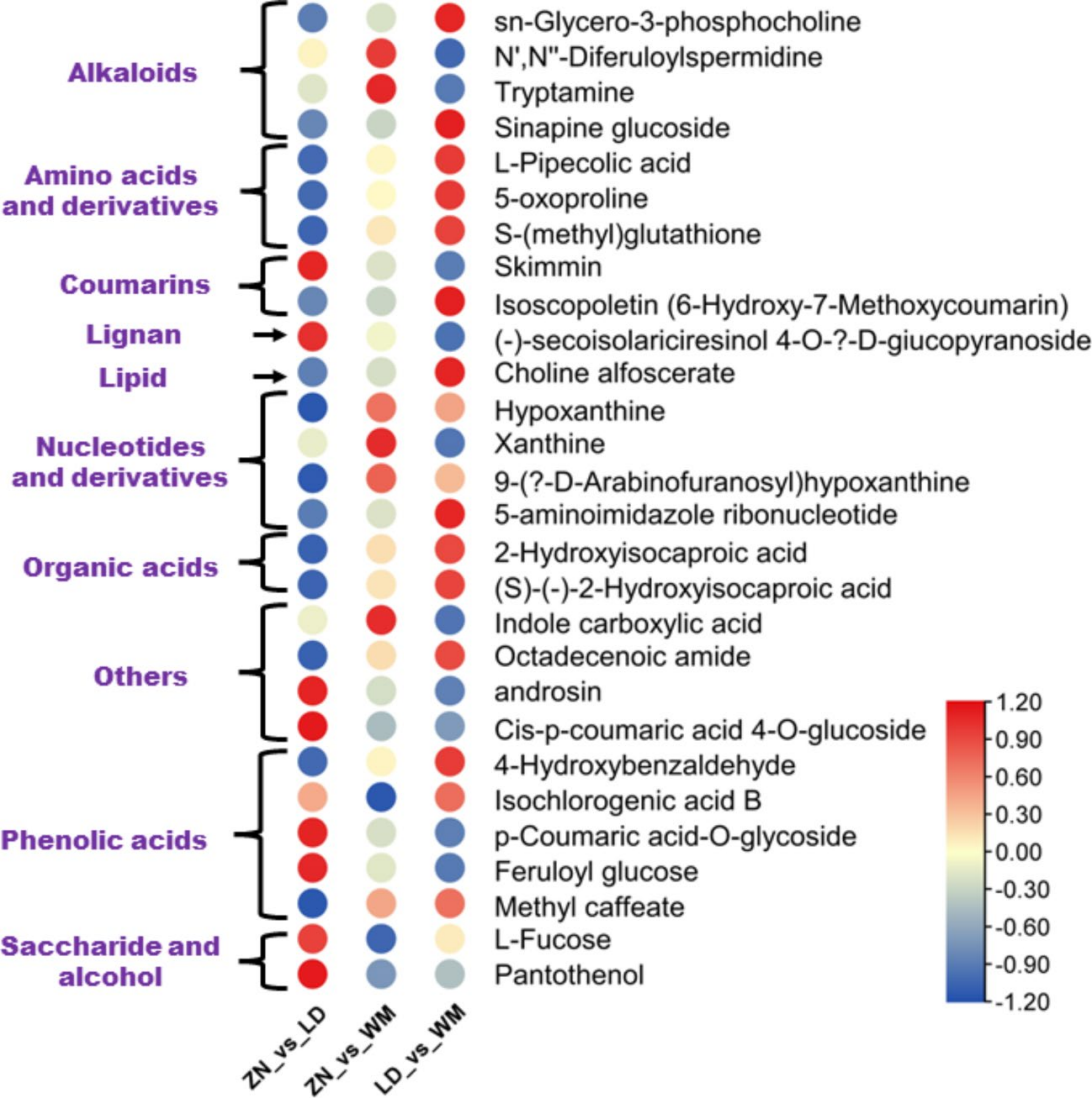
Variation characteristic of the relative content of other DAMs in dragon fruit from the three different altitudes. The scaled Log2 fold change of each metabolite is plotted in a blue-yellow-red color scale. The small red circles indicate the highest accumulation. WM, Wangmo County; LD, Luodian County; ZN, Zhenning County

The accumulation patterns of other DAMs in the dragon fruits from the three different altitudes are shown in Figure7. The results showed that other important bioactive compounds, such as 4-hydroxybenzaldehyde, isochlorogenic acid B and methyl caffeate (phenolic acids), isoscopoletin (coumarin); tryptamine, sn-glycero-3-phosphocholine, and sinapine glucoside (alkaloids); L-pipecolic acid, 5-oxoproline, S-(methyl)glutathione (amino acids and derivatives); 2-hydroxyisocaproic acid and (S)-(-)-2-hydroxyisocaproic acid (organic acids) accumulated highly in fruit from WM than others (Fig. 7). Skimmin, androsin, (-)-secoisolariciresinol 4-*O*-β-D-giucopyranoside, feruloyl glucose, *p*-coumaric acid-*O*-glycoside, L-fucose and pantothenol exhibited the highest content in fruits from LD, followed by WM and ZN (Fig. 7).

## Discussion

Dragon fruit is a promising functional food with tremendous pharmacological abilities against chronic diseases [25, 28, 32–40]. However, the content of bioactive compounds in dragon fruits varies significantly depending on the growing environmental conditions [37, 43]. Thus, the present study investigated changes in the metabolite profiles of *H. polyrhizus.* cv. Jindu1 fruits from three major growing areas in China, mainly different in altitude. The three locations have different altitudes and mostly similar soil and climatic conditions. Our analyses confirmed that the altitude significantly affects the quality values of dragon fruits. We observed a great variation in the relative content of metabolite classes among the fruits from different origins. Fruits from ZN had higher relative terpenoid content. Compared to other locations, fruits from LD showed higher relative content of coumarins and phenolic acids. Fruits from WM showed the highest relative content of flavonoids, alkaloids, amino acids and derivatives, nucleotides and derivatives, and vitamins, indicating that they were prime in quality. These results denote that the geographical conditions at WM (higher altitude) are the most suitable for producing higher nutritional and therapeutical dragon fruits. Concordantly, Naryal et al. found that the weight of apricot fruits decreased with elevation, while the water content increased with the decrease in altitude [48]. It is also demonstrated that the sweetness of blueberry fruits increases with altitude, and fruits from low-altitude areas are more acidic [49]. Taken together, these findings show that cultivating pitaya varieties at high altitudes may produce good taste and marked health-beneficial dragon fruits. Similar findings were reported in blueberry [49]. Betacyanin is the major antioxidant compound in dragon fruit and is used as a natural coloring agent in food products [41, 42]. The metabolomics analysis revealed no difference in betacyanin accumulation, inferring its biosynthesis is less affected by the altitude.

Altitude is a major determinant of fruit quality [16–18]. At high altitudes, the temperature decreases while solar radiation (light intensity, UV light, etc.) increases, resulting in the stimulation of UV-absorbing compounds (mainly flavonoids) biosynthesis and accumulation in plant organs [16, 18, 50]. Herein, we found that the difference in altitude induces variation in metabolite profiles of dragon fruits from the three locations. Sixty-nine significant DAMs were identified across the fruits from the three sites. The classification of DAMs showed that flavonoids were the main DAMs. Supportively, KEGG analysis unveiled that the biosynthesis of isoflavonoid, flavone, and flavonol were the major differently regulated pathways under the three growing conditions. The flavonoid biosynthesis was significantly induced under WM (650 m) environmental conditions, followed by LD (420 m) and ZN (356 m). These findings are consistent with previous reports indicating an enhanced biosynthesis and accumulation of phenylpropanoid pathway-related compounds (flavonoid, anthocyanins, phenolic acids, etc.) in plants at high altitudes [16, 17, 49, 51]. Particularly, Wang et al. found that *Lycium barbarum* fruits from high-altitude locations in China had higher flavonoid content [14]. Furthermore, our results denote that climatic conditions at WM may stimulate signal transduction mechanisms in developing dragon fruits, leading to higher induction of flavonoid biosynthesis structural and regulatory genes’ expressions [51]. Flavonoids are the most diverse class of polyphenol secondary metabolites and the third largest class of natural products broadly distributed in the plant kingdom [52]. Most flavonoid compounds play vital roles in plants’ auto-defense processes against pathogens, plant-environment interactions, ultraviolet (UV) radiation, and abiotic stress tolerance [52, 53]. We then inferred that fruit from WM may resist pathogen attacks during storage more than those from the other locations. It is demonstrated that dragon fruits with higher antioxidants (flavonoids, phenolic acids, vitamin C, betaine, etc.) perform well during postharvest storage [54]. Further studies are required to verify these statements. Besides, many other factors, such as irrigation, water qualities, soils, fertilization, wind direction, photoperiods, and various annual maintenance operations, can also affect the quality characteristics of fruits [19]. The low altitudinal difference between LD and ZN suggests that these factors might also be involved in the observed metabolite profile variation. New studies combining all these environmental factors have to be designed and carefully performed to thoroughly understand the environmental influence on dragon fruit characteristics.

The higher flavonoid content of fruits from WM coupled with the relatively highest content of most of the other differentially accumulated bioactive compounds suggests that they may own very strong pharmacological properties compared to fruits from other localities. The ability of flavonoid compounds to cure or prevent diseases, such as diabetes, cancer, oxidative stress, amnesia, inflammations, microbial infections, and cardiovascular dysfunctions, has been proven [55–61]. For instance, vitexin and isovitexin were the most highly accumulated flavonoids in fruits from WM. These compounds are potential substitute drugs for diverse diseases and have received considerable attention due to their various pharmacological attributes, including antioxidant, anti-hyperalgesic, anti-inflammatory, anti-cancer, neuroprotective, etc [62, 63]. Besides, we identified vitexin and Kaempferol 3-*O*-[2-*O*-*β*-D-galactose-6-*O*-*a*-L-rhamnose]-*β*-D-glucoside as potential biomarkers for discriminating between dragon fruits from different altitudes. Metabolic biomarkers are critical resources used to authenticate, differentiate, and assess the quality of plant-derived products from diverse origins [64]. Isovitexin has been detected as a marker of the Brazilian crude drug extracts from *Echinodorus scaber* and *E. grandifloras* [65]. Further quantifications of these two potential biomarkers in dragon fruits from many genotypes and origins are required to confirm them as discriminatory biomarkers.

## Conclusion

Overall, this study applied metabolomics analysis to compare the accumulation characteristics of nutritional and bioactive compounds in dragon fruits from three altitudes, including LD (420 m), WM (650 m), and ZN (356 m). We found that fruits from WM (highest altitude) were prime in quality, with significantly highest levels of flavonoids, alkaloids, amino acids and derivatives, vitamins and nucleotides and derivatives. Fruits from LD had the significantly highest relative content of phenolic acids and coumarins and ranked second in terms of accumulation of flavonoid compounds. The fruits from ZN (lowest altitude) had the highest relative content of organic acids. We identified DAMs and revealed their accumulation patterns in the fruits from the three origins. The flavonoid biosynthesis pathway was the most affected by changes in altitudes. Furthermore, we uncovered two potential metabolic biomarkers (vitexin and kaempferol 3-*O*-[2-*O*-β-D-galactose-6-*O*-a-L-rhamnose]-β-D-glucoside) that may serve to discriminate between dragon fruits from different origins. Our results show that high-altitude regions may be ideal for producing high nutritional and therapeutic dragon fruits. Moreover, they provide a fundamental basis for further studies toward the environment-based production of dragon fruits for specific purposes.

## Materials and methods

### Plant material and soil chemical properties

This study used *H. polyrhizus* cv. Jindu1 as the experimental material. Jindu1 is the main cultivated pitaya variety in Guizhou province, China, with an area of about 1/4 of the total planting area in the province. Fruit samples were collected from three growing areas, mainly different in altitudes: Wangmo County (WM, 650 m), Luodian County (LD, 420 m), and Zhenning County (ZN, 356 m) (Figure S1). Three plantations at each location were randomly selected, and conventional field management practices were applied. The soil chemical properties at each location are presented in TableS3. The soil properties, including pH, total nitrogen, alkaline hydrolyzed nitrogen, total phosphorus, available phosphorus, organic matter, total potassium, and available potassium contents were evaluated according to the NY/T 1121.2–2006 Soil Testing methods [66, 67]. The fruit samples were collected from five-year-old Jindu1 trees during the peak harvest period in June-July 2022. Approximately 30 fruit pulps from six representative trees were equally mixed to represent one biological replicate in each plantation, and the three biological replicates were made from the three plantations. All nine samples were in-situ frozen in liquid nitrogen and further stored at -80 ^o^C until the UPLC-MS analysis.

### Widely targeted metabolomics analysis of dragon fruits

The pulp samples were freeze-dried and subsequently reduced to powder using a mixer mill (MM 400, Retsch, Haan, Germany). The crushing was operated at 30 Hz for 1.5 min. Then, lyophilized powder (100 mg) of each sample was extracted at 4 °C for 6 h with 1.2 mL of 70% methanol, followed by centrifugation (15 min at 15,000 g). Next, the supernatants were collected and filtrated (0.22 μm micropore membrane, SCAA-104, ANPEL, Shanghai, China). All extracts were stored at -20 ^o^C up to the UPLC-ESI-QqQLIT-MS/MS analysis at MWDB (Metware Biotechnology Co., Ltd., Wuhan, China) [68–70]. Equal volumes of all sample extracts were mixed to constitute quality control (QC) samples. The metabolomics was performed as per previously described methods [69, 70], and detailed information about the liquid phase and MS conditions is presented in TableS3.

### Identification and quantification of metabolites

The qualitative identification of each metabolite was achieved by integrating spectrum, retention time (Rt), and mass spectra information. The Q1 (accurate precursor ions) value, Q3 (product ion) value, Rts, and fragmentation patterns were compared with standards (when they were available). When no standard was available, the metabolites were identified by reference to a local self-built database (Metware Biotechnology Co., Ltd., Wuhan, China) and public databases, including KNApSAcK (a comprehensive species-metabolite relationship database), MassBank (Europe high quality mass spectral database), HMDB (human metabolome database), METLIN (a metabolite mass spectral database), and MoTo DB (metabolome tomato database). All isotope signals were discarded to avoid duplication in the metabolite list [68, 69, 71]. Finally, we carefully checked all identified metabolites through comparison to the phytochemical dictionary (CRC, natural products database) and the literature. All identified metabolite’s relative content was computed via the MRM modes (QqQ MS analysis).

### Statistical analysis

After data quality assessment and standardization via Zscore, we conducted PCA (principal component analysis), K-means, HCA (hierarchical clustering analysis) and OPLS-DA (orthogonal partial least squares discriminant analysis) analyses in R (version 3.5.0, www.r-project.org) using the packages prcomp, cluster package, pheatmap and MetaboAnalystR, respectively. Significant DAMs (differentially accumulated metabolites) were detected using the R-programming language ggplot2 program at thresholds of Log2FC ˃ 1, VIP ≥ 1, and *p*-value < 0.05. The VIP (variable important in projection) values were obtained from the OPLS-DA. The functional annotation of DAMs was achieved through mapping to the KEGG (Kyoto Encyclopedia of Genes and Genomes) database (http://www.kegg.jp/kegg/pathway.html). The metabolite sets enrichment analysis (MSEA) and hypergeometric test were integrated to identify the most enriched pathways. GraphPad Prism (version 9.0.0121) and TBtools software [72] were used for graph construction. ANOVA (analysis of variance) and post hoc test (Tukey test) were performed for multiple comparisons at *P* < 0.05.

### Electronic supplementary material

Below is the link to the electronic supplementary material.

Supplementary Material 1

Supplementary Material 2

## Data Availability

All data generated or analyzed during this study are included in this published article and its supplementary information files. The datasets are available from the corresponding author upon reasonable request.

## Abbreviations

WM: Wangmo County
LD: Luodian County
ZN: Zhenning County
DAM: differentially accumulated metabolites
UPLC: Ultra performance liquid chromatography
QC: Quality control
ESI: Electrospray ionization
HCA: Hierarchical clustering analysis
PCA: Principal component analysis
OPLS-DA: Orthogonal projections to latent structures discriminant analysis
VIP: The variable importance for the projection
