# Supplementary material for: Altitudinal variation of dragon fruit metabolite profiles as revealed by UPLC-MS/MS-based widely targeted metabolomics analysis
Source: BMC Plant Biol. 2024 Apr 29;24:344. doi: 10.1186/s12870-024-05011-w (PMC11057076; doi:10.1186/s12870-024-05011-w)
Supplement: Supplementary file 1 — Supplementary Material 1 [file 12870_2024_5011_MOESM1_ESM.pdf]

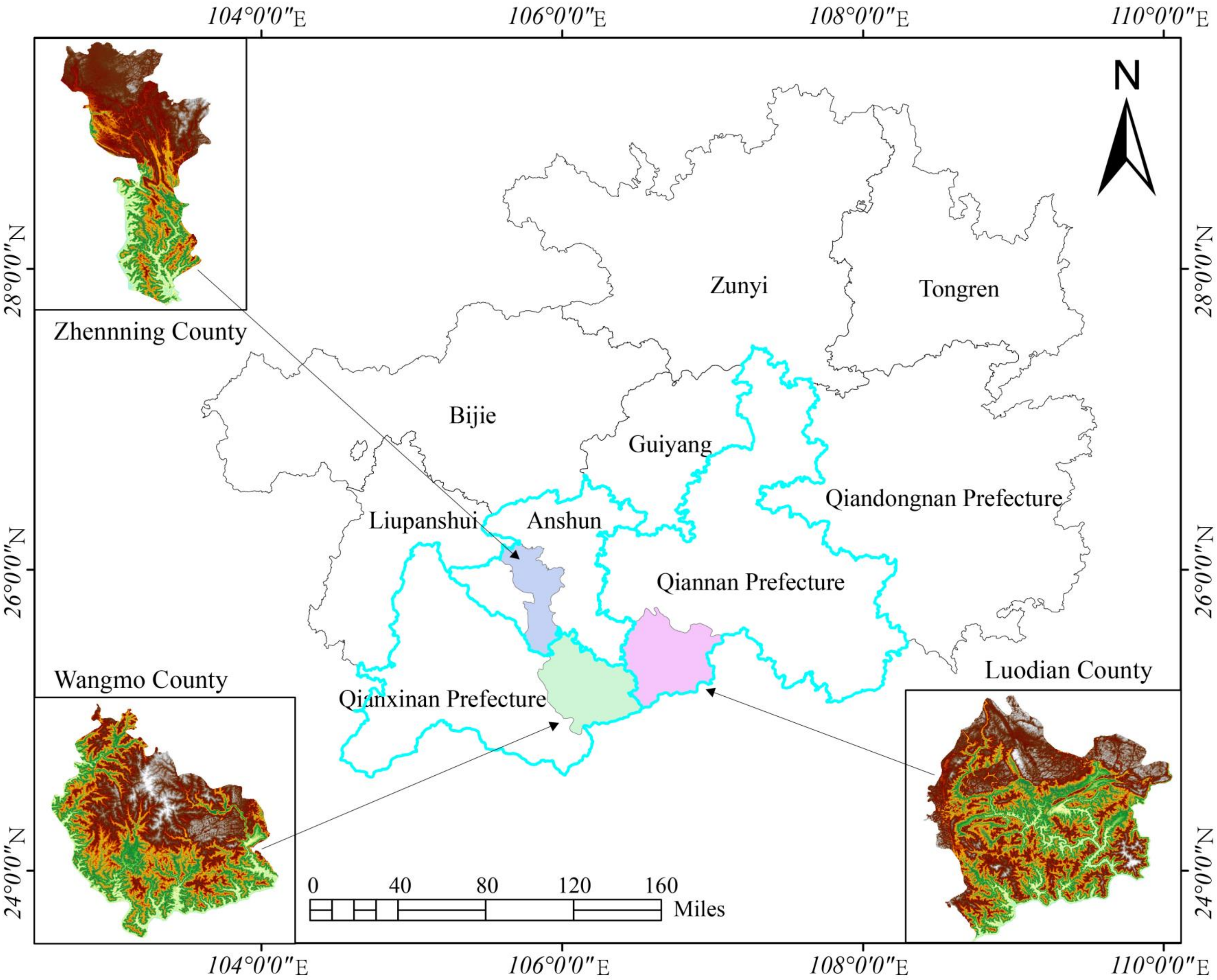

Figure S1. Map of Guizhou province (China) with a spotlight on the three locations of the study areas: Zhenning County (ZN), Wangmo County (WM) and Luodian County (LD).

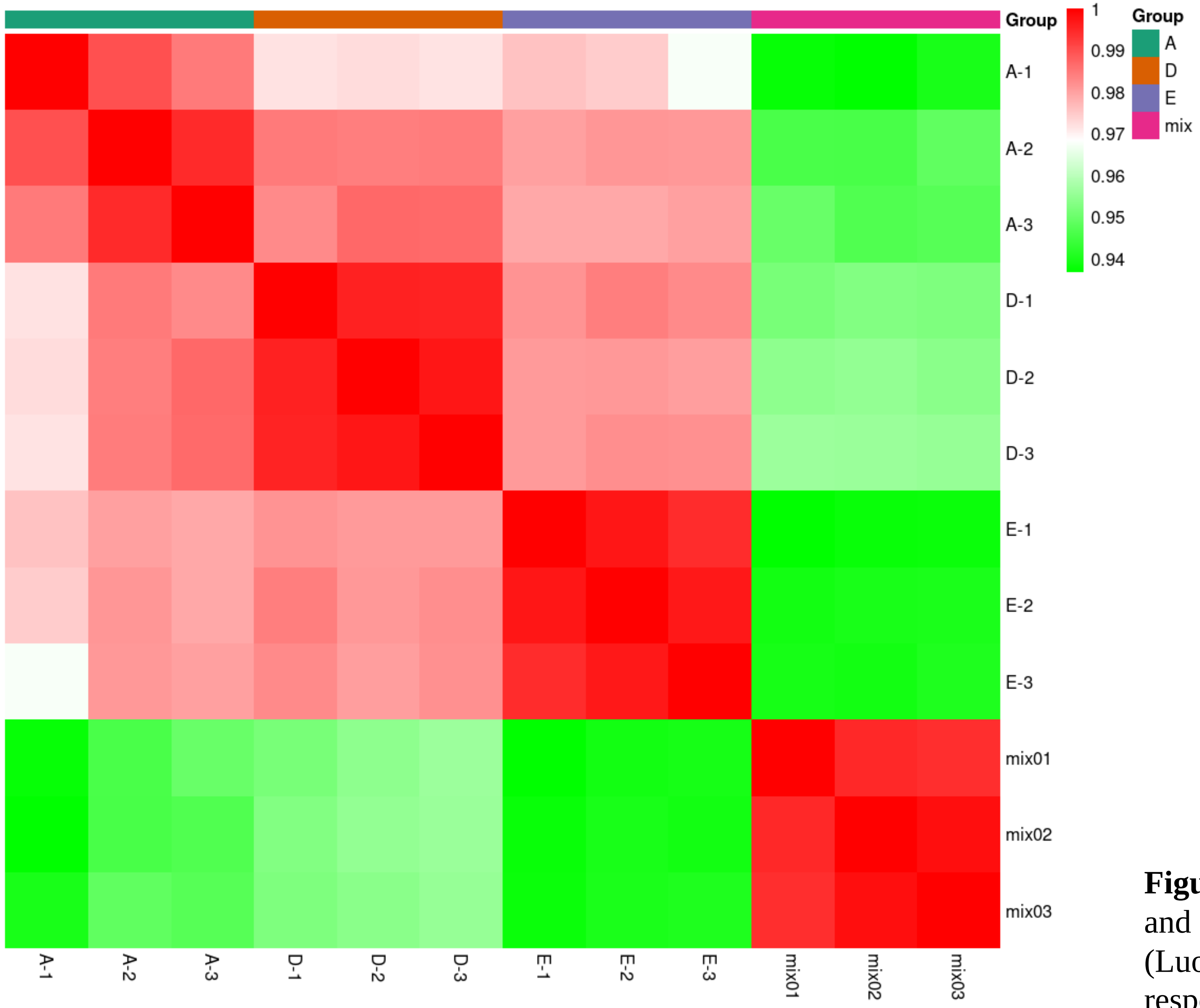

Figure S2. Correlation analysis of samples. A, D, and E indicate ZN (Zhenning County), LD (Luodian County), and WM (Wangmo County), respectively.

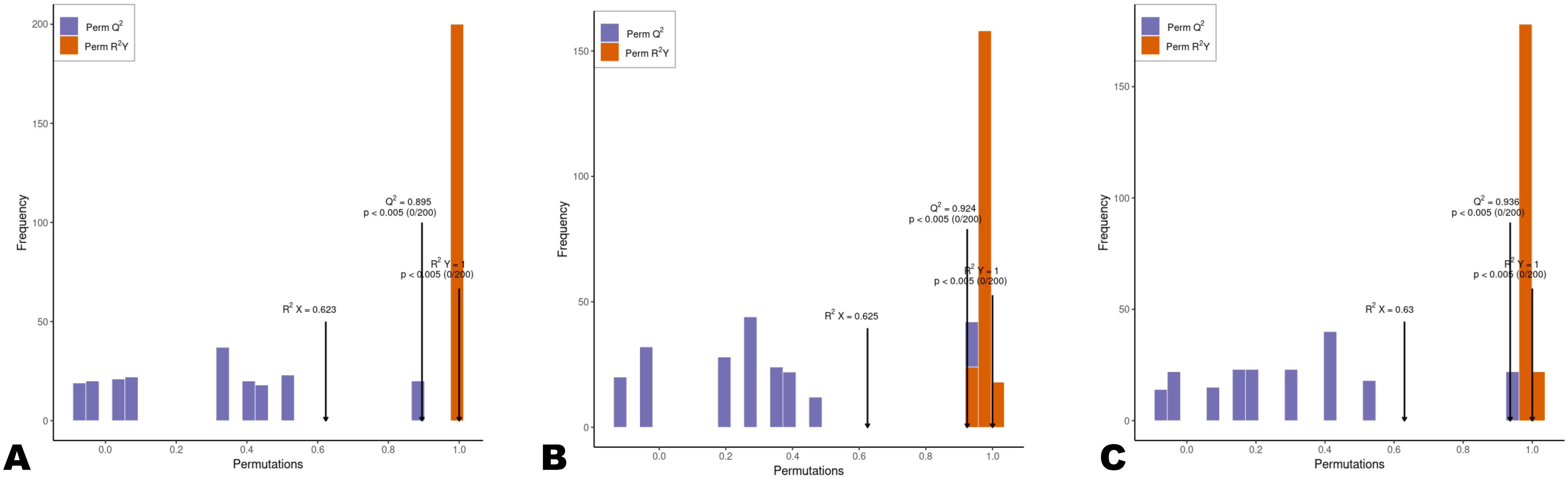

Figure S3. (A)-(C) OPLS-DA analysis results for the pairwise comparison between ZN_vs_LD and ZN_vs_WM, LD_vs_WM, respectively. WM, Wangmo County; LD, Luodian County; ZN, Zhenning County.

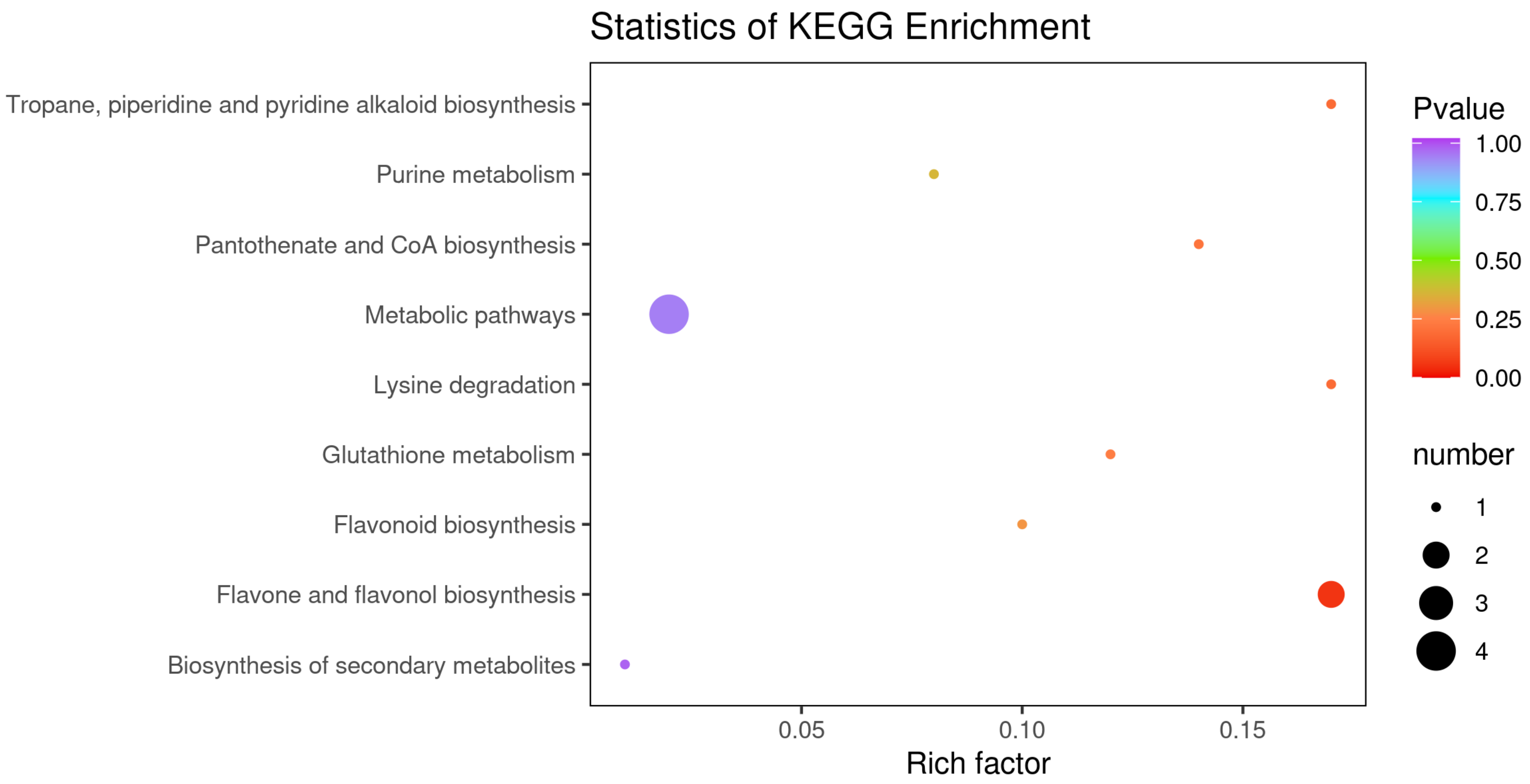

Figure S4. KEGG annotations and enrichment results of the DAMs between LD_vs_WM. WM, Wangmo County; LD, Luodian County

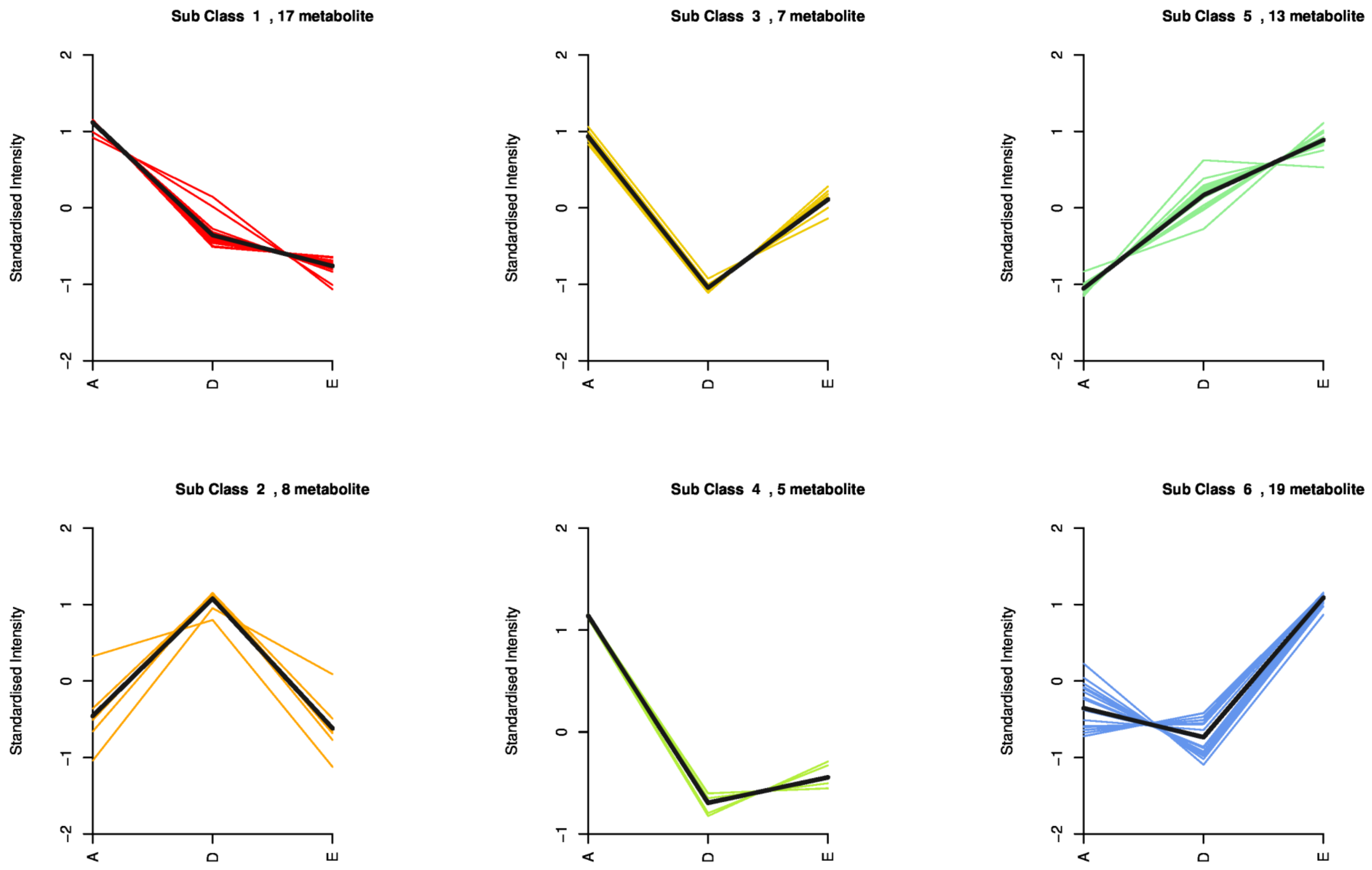

Figure S5. K-means analysis results of all DAMs. A, D, and E indicate ZN (Zhenning County), LD (Luodian County), and WM (Wangmo County), respectively.

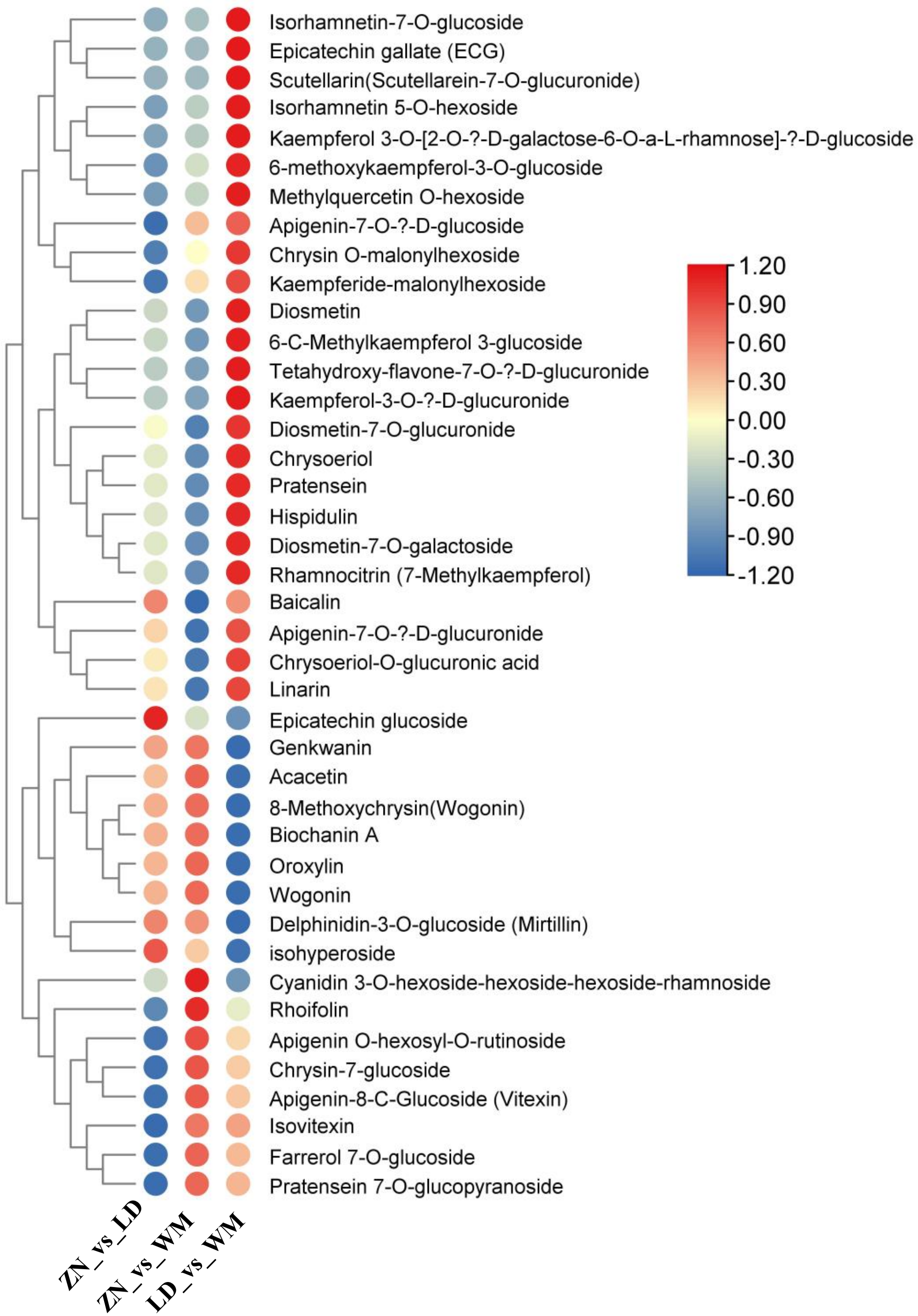

Figure S6. Variation characteristic of the relative content of differentially accumulated flavonoids in dragon fruit from the three different altitudes. WM, Wangmo County; LD, Luodian County; ZN, Zhenning County.
